# Classification of current anticancer immunotherapies

**DOI:** 10.18632/oncotarget.2998

**Published:** 2014-12-18

**Authors:** Lorenzo Galluzzi, Erika Vacchelli, José-Manuel Bravo-San Pedro, Aitziber Buqué, Laura Senovilla, Elisa Elena Baracco, Norma Bloy, Francesca Castoldi, Jean-Pierre Abastado, Patrizia Agostinis, Ron N. Apte, Fernando Aranda, Maha Ayyoub, Philipp Beckhove, Jean-Yves Blay, Laura Bracci, Anne Caignard, Chiara Castelli, Federica Cavallo, Estaban Celis, Vincenzo Cerundolo, Aled Clayton, Mario P. Colombo, Lisa Coussens, Madhav V. Dhodapkar, Alexander M. Eggermont, Douglas T. Fearon, Wolf H. Fridman, Jitka Fučíková, Dmitry I. Gabrilovich, Jérôme Galon, Abhishek Garg, François Ghiringhelli, Giuseppe Giaccone, Eli Gilboa, Sacha Gnjatic, Axel Hoos, Anne Hosmalin, Dirk Jäger, Pawel Kalinski, Klas Kärre, Oliver Kepp, Rolf Kiessling, John M. Kirkwood, Eva Klein, Alexander Knuth, Claire E. Lewis, Roland Liblau, Michael T. Lotze, Enrico Lugli, Jean-Pierre Mach, Fabrizio Mattei, Domenico Mavilio, Ignacio Melero, Cornelis J. Melief, Elizabeth A. Mittendorf, Lorenzo Moretta, Adekunke Odunsi, Hideho Okada, Anna Karolina Palucka, Marcus E. Peter, Kenneth J. Pienta, Angel Porgador, George C. Prendergast, Gabriel A. Rabinovich, Nicholas P. Restifo, Naiyer Rizvi, Catherine Sautès-Fridman, Hans Schreiber, Barbara Seliger, Hiroshi Shiku, Bruno Silva-Santos, Mark J. Smyth, Daniel E. Speiser, Radek Spisek, Pramod K. Srivastava, James E. Talmadge, Eric Tartour, Sjoerd H. Van Der Burg, Benoît J. Van Den Eynde, Richard Vile, Hermann Wagner, Jeffrey S. Weber, Theresa L. Whiteside, Jedd D. Wolchok, Laurence Zitvogel, Weiping Zou, Guido Kroemer

**Affiliations:** ^1^ Equipe 11 labellisée pas la Ligue Nationale contre le Cancer, Centre de Recherche des Cordeliers, Paris, France; ^2^ INSERM, U1138, Paris, France; ^3^ Gustave Roussy Cancer Campus, Villejuif, France; ^4^ Université Paris Descartes/Paris V, Sorbonne Paris Cité, Paris, France; ^5^ Faculté de Medicine, Université Paris Sud/Paris XI, Le Kremlin-Bicêtre, France; ^6^ Sotio a.c., Prague, Czech Republic; ^7^ Pole d'innovation thérapeutique en oncologie, Institut de Recherches Internationales Servier, Suresnes, France; ^8^ Cell Death Research and Therapy (CDRT) Laboratory, Dept. of Cellular and Molecular Medicine, University of Leuven, Leuven, Belgium; ^9^ The Shraga Segal Dept. of Microbiology, Immunology and Genetics, Faculty of Health Sciences, Ben-Gurion University of the Negev, Beer-Sheva, Israel; ^10^ Group of Immune receptors of the Innate and Adaptive System, Institut d'Investigacions Biomédiques August Pi i Sunyer (IDIBAPS), Barcelona, Spain; ^11^ INSERM, U1102, Saint Herblain, France; ^12^ Institut de Cancérologie de l'Ouest, Saint Herblain, France; ^13^ Translational Immunology Division, German Cancer Research Center, Heidelberg, Germany; ^14^ Equipe 11, Centre Léon Bérard (CLR), Lyon, France; ^15^ Centre de Recherche en Cancérologie de Lyon (CRCL), Lyon, France; ^16^ Dept. of Hematology, Oncology and Molecular Medicine, Istituto Superiore di Sanità, Rome, Italy; ^17^ INSERM, U1160, Paris, France; ^18^ Groupe Hospitalier Saint Louis-Lariboisière - F. Vidal, Paris, France; ^19^ Unit of Immunotherapy of Human Tumors, Dept. of Experimental Oncology and Molecular Medicine, Fondazione IRCCS Istituto Nazionale Tumori, Milano, Italy; ^20^ Molecular Biotechnology Center, Dept. of Molecular Biotechnology and Health Sciences, University of Torino, Torino, Italy; ^21^ Cancer Immunology, Inflammation and Tolerance Program, Georgia Regents University Cancer Center, Augusta, GA, USA; ^22^ MRC Human Immunology Unit, Weatherall Institute of Molecular Medicine, University of Oxford, Oxford, UK; ^23^ Institute of Cancer & Genetics, School of Medicine, Cardiff University, Cardiff, UK; ^24^ Velindre Cancer Centre, Cardiff, UK; ^25^ Knight Cancer Institute, Oregon Health & Science University, Portland, OR, USA; ^26^ Sect. of Hematology and Immunobiology, Yale Cancer Center, Yale University, New Haven, CT, USA; ^27^ Cold Spring Harbor Laboratory, Cold Spring Harbor, NY, USA; ^28^ Université Pierre et Marie Curie/Paris VI, Paris, France; ^29^ Equipe 13, Centre de Recherche des Cordeliers, Paris, France; ^30^ Dept. of Immunology, 2nd Faculty of Medicine and University Hospital Motol, Charles University, Prague, Czech Republic; ^31^ Dept. of Pathology and Laboratory Medicine, Perelman School of Medicine, University of Pennsylvania, Philadelphia, PA, USA; ^32^ Laboratory of Integrative Cancer Immunology, Centre de Recherche des Cordeliers, Paris, France; ^33^ INSERM, UMR866, Dijon, France; ^34^ Centre Georges François Leclerc, Dijon, France; ^35^ Université de Bourgogne, Dijon, France; ^36^ Center for Cancer Research, National Cancer Institute (NCI), National Institutes of Health (NIH), Bethesda, MD, USA; ^37^ Lombardi Comprehensive Cancer Center, Georgetown University, Washington, DC, USA; ^38^ Dept. of Microbiology and Immunology, Sylvester Comprehensive Cancer Center, University of Miami, Miller School of Medicine, Miami, FL, USA; ^39^ Sect. of Hematology/Oncology, Immunology, Tisch Cancer Institute, Icahn School of Medicine at Mount Sinai, New York, NY, USA; ^40^ Glaxo Smith Kline, Cancer Immunotherapy Consortium, Collegeville, PA, USA; ^41^ INSERM, U1016, Paris, France; ^42^ CNRS, UMR8104, Paris, France; ^43^ Hôpital Cochin, AP-HP, Paris, France; ^44^ National Center for Tumor Diseases, University Medical Center Heidelberg, Heidelberg, Germany; ^45^ Dept. of Surgery, University of Pittsburgh, Pittsburgh, PA, USA; ^46^ University of Pittsburgh Cancer Institute, Hillman Cancer Center, Pittsburgh, PA, USA; ^47^ Dept. of Immunology and Infectious Diseases and Microbiology, University of Pittsburgh, Pittsburgh, PA, USA; ^48^ Dept. of Microbiology, Tumor and Cell Biology, Karolinska Institute, Stockholm, Sweden; ^49^ Metabolomics and Cell Biology Platforms, Gustave Roussy Cancer Campus, Villejuif, France; ^50^ Dept. of Oncology, Karolinska Institute Hospital, Stockholm, Sweden; ^51^ University of Pittsburgh Cancer Institute Laboratory, Pittsburgh, PA, USA; ^52^ National Center for Cancer Care and Research, Hamad Medical Corporation, Doha, Qatar; ^53^ Academic Unit of Inflammation and Tumour Targeting, Dept. of Oncology, University of Sheffield Medical School, Sheffield, UK; ^54^ INSERM, UMR1043, Toulouse, France; ^55^ CNRS, UMR5282, Toulouse, France; ^56^ Laboratoire d'Immunologie, CHU Toulouse, Université Toulouse II, Toulouse, France; ^57^ Unit of Clinical and Experimental Immunology, Humanitas Clinical and Research Institute, Rozzano, Italy; ^58^ Dept. of Biochemistry, University of Lausanne, Epalinges, Switzerland; ^59^ Dept. of Medical Biotechnologies and Translational Medicine, University of Milan, Rozzano, Italy; ^60^ Dept. of Immunology, Centro de Investigación Médica Aplicada (CIMA), Universidad de Navarra, Pamplona, Spain; ^61^ Dept. of Oncology, Clínica Universidad de Navarra, Pamplona, Spain; ^62^ ISA Therapeutics, Leiden, The Netherlands; ^63^ Dept. of Immunohematology and Blood Transfusion, Leiden University Medical Center, Leiden, The Netherlands; ^64^ Research Dept. of Surgical Oncology, The University of Texas, MD Anderson Cancer Center, Houston, TX, USA; ^65^ Istituto Giannina Gaslini, Genova, Italy; ^66^ Center for Immunotherapy, Roswell Park Cancer Institute, Buffalo, NY, USA; ^67^ Dept. of Neurological Surgery, University of California San Francisco, San Francisco, CA, USA; ^68^ The Jackson Laboratory for Genomics Medicine, Farmington, CT, USA; ^69^ Div. of Hematology/Oncology, Northwestern University, Feinberg School of Medicine, Chicago, IL, USA; ^70^ The James Buchanan Brady Urological Institute, The Johns Hopkins Medical Institutions, Baltimore, MD, USA; ^71^ Lankenau Institute for Medical Research, Wynnewood, PA, USA; ^72^ Dept. of Pathology, Anatomy and Cell Biology, Sidney Kimmel Medical College, Philadelphia, PA, USA; ^73^ Cell Biology and Signaling Program, Kimmel Cancer Center, Thomas Jefferson University, Philadelphia, PA, USA; ^74^ Laboratorio de Inmunopatología, Instituto de Biología y Medicina Experimental (IBYME), Buenos Aires, Argentina; ^75^ National Cancer Institute (NCI), National Institutes of Health (NIH), Bethesda, MD, USA; ^76^ Memorial Sloan Kettering Cancer Center (MSKCC), New York, NY, USA; ^77^ Dept. of Pathology, The Cancer Research Center, The University of Chicago, Chicago, IL, USA; ^78^ Institute of Medical Immunology, Martin Luther University Halle-Wittenberg, Halle, Germany; ^79^ Dept. of Immuno-GeneTherapy, Mie University Graduate School of Medicine, Tsu, Japan; ^80^ Instituto de Medicina Molecular, Universidade de Lisboa, Lisboa, Portugal; ^81^ Immunology in Cancer and Infection Laboratory, QIMR Berghofer Medical Research Institute, Herston, Queensland, Australia; ^82^ School of Medicine, University of Queensland, Herston, Queensland, Australia; ^83^ Dept. of Oncology, University of Lausanne, Lausanne, Switzerland; ^84^ Ludwig Cancer Research Center, Lausanne, Switzerland; ^85^ Dept. of Immunology, University of Connecticut School of Medicine, Farmington, CT, USA; ^86^ Carole and Ray Neag Comprehensive Cancer Center, Farmington, CT, USA; ^87^ Laboratory of Transplantation Immunology, Dept. of Pathology and Microbiology, University of Nebraska Medical Center, Omaha, NE, USA; ^88^ INSERM, U970, Paris, France; ^89^ Paris-Cardiovascular Research Center (PARCC), Paris, France; ^90^ Service d'Immunologie Biologique, Hôpital Européen Georges Pompidou (HEGP), AP-HP, Paris, France; ^91^ Dept. of Clinical Oncology, Leiden University Medical Center, Leiden, The Netherlands; ^92^ Ludwig Institute for Cancer Research, Brussels, Belgium; ^93^ de Duve Institute, Brussels, Belgium; ^94^ Université Catholique de Louvain, Brussels, Belgium; ^95^ Dept. of Molecular Medicine and Immunology, Mayo Clinic College of Medicine, Rochester, MN, USA; ^96^ Institute of Medical Microbiology, Immunology and Hygiene, Technical University Munich, Munich, Germany; ^97^ Donald A. Adam Comprehensive Melanoma Research Center, Moffitt Cancer Center, Tampa, FL, USA; ^98^ University of Pittsburgh School of Medicine, Pittsburgh, PA, USA; ^99^ Dept. of Medicine and Ludwig Center, Memorial Sloan Kettering Cancer Center (MSKCC), New York, NY, USA; ^100^ Weill Cornell Medical College, New York, NY, USA; ^101^ INSERM, U1015, Villejuif, France; ^102^ Centre d'Investigation Clinique Biothérapie 507 (CICBT507), Gustave Roussy Cancer Campus, Villejuif, France; ^103^ University of Michigan, School of Medicine, Ann Arbor, MI, USA; ^104^ Pôle de Biologie, Hôpital Européen Georges Pompidou (HEGP), AP-HP, Paris, France

**Keywords:** adoptive cell transfer, checkpoint blockers, dendritic cell-based interventions, DNA-based vaccines, immunostimulatory cytokines, peptide-based vaccines, oncolytic viruses, Toll-like receptor agonists

## Abstract

During the past decades, anticancer immunotherapy has evolved from a promising therapeutic option to a robust clinical reality. Many immunotherapeutic regimens are now approved by the US Food and Drug Administration and the European Medicines Agency for use in cancer patients, and many others are being investigated as standalone therapeutic interventions or combined with conventional treatments in clinical studies. Immunotherapies may be subdivided into “passive” and “active” based on their ability to engage the host immune system against cancer. Since the anticancer activity of most passive immunotherapeutics (including tumor-targeting monoclonal antibodies) also relies on the host immune system, this classification does not properly reflect the complexity of the drug-host-tumor interaction. Alternatively, anticancer immunotherapeutics can be classified according to their antigen specificity. While some immunotherapies specifically target one (or a few) defined tumor-associated antigen(s), others operate in a relatively non-specific manner and boost natural or therapy-elicited anticancer immune responses of unknown and often broad specificity. Here, we propose a critical, integrated classification of anticancer immunotherapies and discuss the clinical relevance of these approaches.

## INTRODUCTION

Our perception of cancer has changed dramatically during the past 3 decades. For instance, it has been appreciated that tumors are not a purely clonal disorder, although in some cases they do evolve from a single (pre-)malignant cell [1-3]. It is now clear that established neoplasms do not consist only of transformed cells, but contain an abundant and heterogeneous non-transformed component, including stromal, endothelial and immune cells [4-6]. We no longer consider the metabolism of cancer cells as completely distinct from that of their normal counterparts [7-9]. We have shown that the survival of transformed cells can critically depend on adaptive responses that *per se* are non-tumorigenic, establishing the concept of non-oncogene addiction [10, 11]. We discovered mechanisms other than intrinsic apoptosis that may be harnessed for therapeutic applications, such as several forms of regulated necrosis [12-14]. Finally, we obtained evidence indicating that the host immune system can recognize (and sometimes react against) (pre-)malignant cells as they transform, proliferate, evolve and respond to therapy, founding the theoretical grounds of anticancer immunosurveillance [15-17]. These conceptual shifts have profound therapeutic implications, some of which have already been translated into clinical realities. For instance, several anticancer agents that are now approved by the US Food and Drug Administration (FDA) and European Medicines Agency (EMA) for use in cancer patients inhibit tumor-associated angiogenesis, perhaps the best characterized interaction between malignant and non-malignant components of the tumor microenvironment [18, 19].

**Figure 1:**
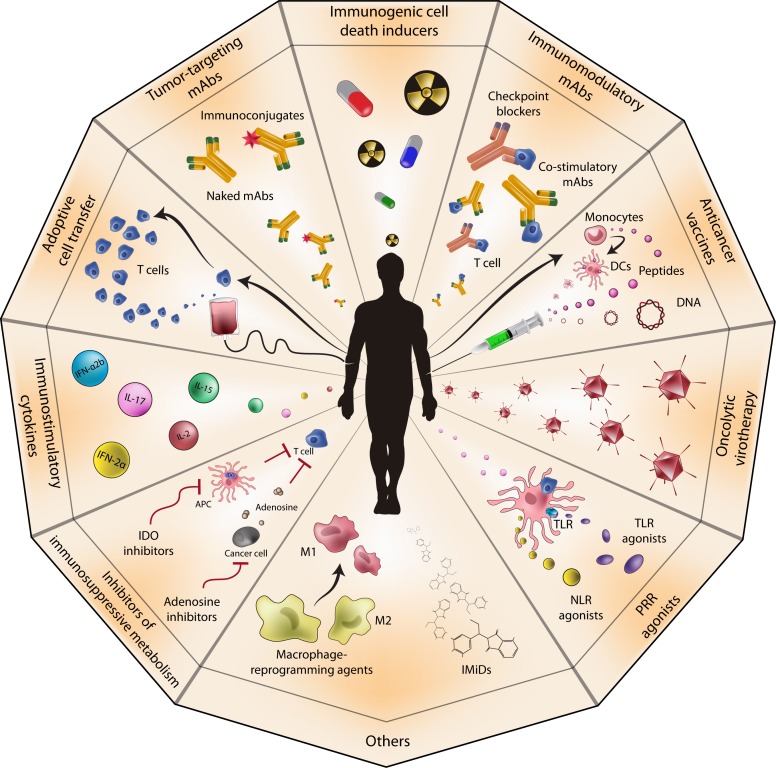
Anticancer immunotherapy Several anticancer immunotherapeutics have been developed during the last three decades, including tumor-targeting and immunomodulatory monoclonal antibodies (mAbs); dendritic cell (DC)-, peptide- and DNA-based anticancer vaccines; oncolytic viruses; pattern recognition receptor (PRR) agonists; immunostimulatory cytokines; immunogenic cell death inducers; inhibitors of immunosuppressive metabolism; and adoptive cell transfer. 1MT, 1-methyltryptophan; APC, antigen-presenting cell; IDO, indoleamine 2,3-dioxigenase; IFN, interferon; IL, interleukin; IMiD, immunomodulatory drug; NLR, NOD-like receptor; TLR, Toll-like receptor.

Over the last decade, great efforts have been dedicated to the development of interventions that mediate antineoplastic effects by initiating a novel or boosting an existing immune response against neoplastic cells (Table 1) [20-32]. This intense wave of preclinical and clinical investigation culminated with the approval of various immunotherapeutic interventions for use in humans (Table 2). In 2013, the extraordinary clinical success of immunotherapy was acknowledged by the Editors of Science Magazine with the designation of “Breakthrough of the Year” [33]. Nonetheless, we have just begun to unravel the therapeutic possibilities offered by anticancer immunotherapy. Clinical studies are being initiated at an ever accelerating pace to test the safety and efficacy of various immunotherapeutic regimens in cancer patients, either as standalone interventions or combined with other antineoplastic agents [34]. The hopes generated by this approach are immense, and several other forms of immunotherapy are expected to obtain regulatory approval within the next few years (Figure 1).

**Table 1:** Currently available anticancer immunotherapies

Paradigm	Licensed*
Tumor-targeting mAbs	YES
Adoptive cell transfer	NO
Oncolytic viruses	YES
DC-based interventions	YES
DNA-based vaccines	NO
Peptide-based vaccines	YES
Immunostimulatory cytokines	YES
Immunomodulatory mAbs	YES
Inhibitors of immunosuppressive metabolism	NO
PRR agonists	YES
ICD inducers	YES
Others	YES

Abbreviations. ICD, immunogenic cell death; DC, dendritic cell; mAb, monoclonal antibody; PRR, pattern recognition receptor.

* in one of its forms for use in cancer patients, by the US Food and Drug Administration or equivalent regulatory agency worldwide.

Anticancer immunotherapies are generally classified as “passive” or “active” based on their ability to (re-)activate the host immune system against malignant cells [35]. From this standpoint, tumor-targeting monoclonal antibodies (mAbs) and adoptively transferred T cells (among other approaches) are considered passive forms of immunotherapy, as they are endowed with intrinsic antineoplastic activity [23, 24, 36, 37]. Conversely, anticancer vaccines and checkpoint inhibitors exert anticancer effects only upon the engagement of the host immune system, constituting clear examples of active immunotherapy [22, 27, 28, 32, 38]. An alternative classification of immunotherapeutic anticancer regimens is based on antigen-specificity. Thus, while tumor-targeting mAbs are widely considered antigen-specific interventions, immunostimulatory cytokines or checkpoint blockers activate anticancer immune responses of unknown (and generally broad) specificity [27, 39-42]. Herein, we critically revise these classifications while discussing the clinical relevance of various forms of anticancer immunotherapy.

**Table 2:** Anticancer immunotherapeutics currently approved by regulatory agencies worldwide

Paradigm	Agent	Indication(s)	Year*	Proposed mechanism of action
Dendritic cell-based immunotherapies	Sipuleucel-T	Prostate carcinoma	2010	Priming of a PAP-specific immune response
Immunogenic cell death inducers	Bleomycin	Multiple hematological and solid tumors	<1995	DNA-damaging agent
	Bortezomib	Mantle cell lymphoma Multiple myeloma	2003	Proteasomal inhibitor
	Cyclophosphamide	Multiple hematological and solid tumors	<1995	Alkylating agent
	Doxorubicin	Multiple hematological and solid tumors	<1995	DNA-intercalating agent
	Epirubicin	Breast carcinoma	1999	DNA-intercalating agent
	Mitoxantrone	Acute myeloid leukemia Prostate carcinoma	<1995	DNA-intercalating agent
	Oxaliplatin	Colorectal carcinoma	2002	DNA-damaging agent
	Photodynamic therapy	Multiple hematological and solid tumors	1996	Induction of oxidative stress with damage to (intra)cellular membranes
	Radiation therapy	Multiple hematological and solid tumors	<1995	DNA-damaging agent and oxidative stress inducer
Immunostimulatory cytokines	IL-2	Melanoma Renal cell carcinoma	<1995	Non-specific immunostimulation
	IFN-α2a	Chronic myeloid leukemia Hairy cell leukemia Melanoma	1999	Non-specific immunostimulation
	IFN-α2b	Multiple hematological and solid tumors	<1995	Non-specific immunostimulation
Immunomodulatory mAbs	Ipilimumab	Melanoma	2011	Blockage of CTLA4-dependent immunological checkpoints
	Nivolumab	Melanoma	2014	Blockage of PDCD1-dependent immunological checkpoints
	Pembrolizumab	Melanoma	2014	Blockage of PDCD1-dependent immunological checkpoints
Oncolytic viruses	Oncorine H101	Head and neck cancer	2005	Selective lysis of malignant cells
Peptide-based vaccines	Vitespen	Renal cell carcinoma	2008	Activation of a tumor-specific immune response
PRR agonists	Bacillus Calmette-Guérin	Non-invasive bladder transitional cell carcinoma	<1995	TLR2/TLR4 agonist
	Imiquimod	Actinic keratosis Condylomata acuminata Superficial basal cell carcinoma	1997	TLR7 agonist
	Mifamurtide	Osteosarcoma	2009	NOD2 agonist
	Monophosphoryl lipid A	Prevention of HPV-associated cervical carcinoma	2009	TLR2/TLR4 agonist
	Picibanil	Gastric carcinoma Head and neck cancer Lung carcinoma Thyroid carcinoma	<1995	TLR2/TLR4 agonist
Tumor-targeting mAbs	Alemtuzumab	Chronic lymphocytic leukemia	2001	Selective recognition/opsonization of CD52^+^ neoplastic cells
	Bevacizumab	Colorectal carcinoma Glioblastoma multiforme Cervical carcinoma Lung carcinoma Renal cell carcinoma	2004	VEGFA neutralization
	Brentuximab vedotin	Anaplastic large cell lymphoma Hodgkin's lymphoma	2011	Selective delivery of MMAE to CD30^+^ neoplastic cells
	Blinatumumab	Acute lymphoblastic leukemia	2014	CD3- and CD19-specific BiTE
	Catumaxomab	Malignant ascites in patients with EPCAM^+^ cancer	2009	CD3- and EPCAM-specific BiTE
	Cetuximab	Head and neck cancer Colorectal carcinoma	2004	Inhibition of EGFR signaling
	Denosumab	Breast carcinoma Prostate carcinoma Bone giant cell tumors	2011	Inhibition of RANKL signaling
	Gemtuzumab ozogamicin	Acute myeloid leukemia	2000	Selective delivery of calicheamicin to CD33^+^ neoplastic cells
	Ibritumomab tiuxetan	Non-Hodgkin lymphoma	2002	Selective delivery of ^90^Y or ^111^In to CD20^+^ neoplastic cells
	Panitumumab	Colorectal carcinoma	2006	Inhibition of EGFR signaling
	Pertuzumab	Breast carcinoma	2012	Inhibition of HER2 signaling
	Obinutuzumab	Chronic lymphocytic leukemia	2013	Selective recognition/opsonization of CD20^+^ neoplastic cells
	Ofatumumab	Chronic lymphocytic leukemia	2009	Selective recognition/opsonization of CD20^+^ neoplastic cells
	Ramucirumab	Gastric or gastroesophageal junction adenocarcinoma	2014	Inhibition of KDR signaling
	Rituximab	Chronic lymphocytic leukemia Non-Hodgkin lymphoma	1997	Selective recognition/opsonization of CD20^+^ neoplastic cells
	Siltuximab	Multicentric Castleman's disease	2014	IL-6 neutralization
	Tositumomab	Non-Hodgkin lymphoma	2003	Selective recognition/opsonization of, or selective delivery of ^90^Y or ^111^In to, CD20^+^ neoplastic cells
	Trastuzumab	Breast carcinoma Gastric or gastroesophageal junction adenocarcinoma	1998	Selective recognition/opsonization of, or selective delivery of mertansine to, HER2^+^ cancer cells
Others	Lenalidomide	Mantle cell lymphoma Myelodysplastic syndrome Multiple myeloma	2005	IKZF degradation and immunomodulation
	Pomalidomide	Multiple myeloma	2013	IKZF degradation and immunomodulation
	Thalidomide	Multiple myeloma	2006	IKZF degradation and immunomodulation
	Trabectedin	Soft tissue sarcoma Ovarian carcinoma	2007	Reprogramming of tumor-associated macrophages

Abbreviations: ACPP, acid phosphatase, prostate; BiTE, Bispecific T-cell engager; CTLA4, cytotoxic T lymphocyte-associated protein 4; EGFR, epidermal growth factor receptor; EPCAM, epithelial cell adhesion molecule; HPV, human papillomavirus; IL, interleukin; IKZF, IKAROS family zinc finger; KDR, kinase insert domain receptor; mAb, monoclonal antibody; MMAE, monomethyl auristatin E; NOD2, nucleotide-binding oligomerization domain containing 2; PDCD1, programmed cell death 1; PRR, pattern recognition receptor; RANKL, Receptor activator of NF-κB ligand; TLR, Toll-like receptor; VEGFA, vascular endothelial growth factor A.

* year of first approval.

### Passive immunotherapy

#### Tumor-targeting mAbs

Tumor-targeting mAbs are the best-characterized form of anticancer immunotherapy, and perhaps the most widely employed in the clinic [43-46]. The expression “tumor-targeting” refers to mAbs that (1) specifically alter the signaling functions of receptors expressed on the surface of malignant cells [47-49]; (2) bind to, and hence neutralize, trophic signals produced by malignant cells or by stromal components of neoplastic lesions [50, 51]; (3) selectively recognize cancer cells based on the expression of a “tumor-associated antigen” (TAA), i.e., an antigen specifically (or at least predominantly) expressed by transformed cells but not (or at least less so) by their non-malignant counterparts [30, 52]. Tumor-targeting mAbs exist in at least 5 functionally distinct variants. First, naked mAbs that inhibit signaling pathways required for the survival or progression of neoplastic cells, but not of their non-malignant counterparts, such as the epidermal growth factor receptor (EGFR)-specific mAb cetuximab, which is approved by the US FDA for the treatment of head and neck cancer (HNC) and colorectal carcinoma (CRC) [47, 48, 53]. Second, naked mAbs that activate potentially lethal receptors expressed on the surface of malignant cells, but not of their non-transformed counterparts, such as tigatuzumab (CS-1008), a mAb specific for tumor necrosis factor receptor superfamily, member 10B, (TNFRSF10B, best known as TRAILR2 or DR5) that is currently under clinical development [49, 54]. Third, immune conjugates, i.e., TAA-specific mAbs coupled to toxins or radionuclides, such as gemtuzumab ozogamicin, an anti-CD33 calicheamicin conjugate currently approved for use in acute myeloid leukemia patients [55, 56]. Fourth, naked TAA-specific mAbs that opsonize cancer cells and hence activate antibody-dependent cell-mediated cytotoxicity (ADCC) [44, 57-59], antibody-dependent cellular phagocytosis [60], and complement-dependent cytotoxicity [61], such as the CD20-specific mAb rituximab, which is currently approved for the treatment of chronic lymphocytic leukemia (CLL) and non-Hodgkin lymphoma [62, 63]. Fifth, so-called “bispecific T-cell engagers” (BiTEs), *i.e.*, chimeric proteins consisting of two single-chain variable fragments from distinct mAbs, one targeting a TAA and one specific for a T-cell surface antigen (*e.g.*, blinatumomab, a CD19- and CD3 BiTE recently approved for the therapy of Philadelphia chromosome-negative precursor B-cell acute lymphoblastic leukemia) [64-69].

The therapeutic activity of opsonizing mAbs and BiTEs clearly relies on the host immune system, implying that these molecules should be considered active immunotherapeutics. Conversely, tumor-targeting mAbs of the first two classes are endowed with intrinsic antineoplastic activity, and have been considered for a long time as passive forms of immunotherapy. However, growing evidence indicates that the actual antineoplastic potential of these molecules does not simply reflect their direct tumor-inhibitory activity, but also involves (at least to some degree) the activation of an anticancer immune response. For instance, cetuximab does not only inhibit EGFR signaling [53], but also promotes ADCC [70], and mediates immunostimulatory effects [71, 72]. Similarly, bevacizumab, a vascular endothelial growth factor A (VEGFA)-neutralizing mAb approved for the treatment of glioblastoma multiforme, CRC, as well as cervical carcinoma, renal cell carcinoma (RCC) and lung carcinoma, not only exerts anti-angiogenic effects [50, 73], but also boosts tumor infiltration by B and T lymphocytes, [74, 75], while inhibiting CD4^+^CD25^+^FOXP3^+^ regulatory T cells (Tregs) [76]. Moreover, polymorphisms in the genes coding for the receptors mainly responsible for ADCC, *i.e.*, Fc fragment of IgG, low affinity IIa, receptor (FCGR2A, also known as CD32) and FCGR3A (also known as CD16a), have been shown to influence the response of cancer patients to most tumor-targeting mAbs [77]. Thus, it is possible (although not formally demonstrated) that tumor-targeting mAbs operate as active immunotherapeutics. Irrespective of this possibility, 18 distinct tumor-targeting mAbs are currently approved by the US FDA for use in cancer patients (source http://www.fda.gov) [45, 46], demonstrating the extraordinary success of this immunotherapeutic paradigm.

#### Adoptive cell transfer

The term “adoptive cell transfer” (ACT) refers to a particular variant of cell-based anticancer immunotherapy that generally involves: (1) the collection of circulating or tumor-infiltrating lymphocytes; (2) their selection/modification/expansion/activation *ex vivo*; and (3) their (re-)administration to patients, most often after lymphodepleting pre-conditioning and in combination with immunostimulatory agents [23, 24, 78-80]. Other anticancer (immune)therapies involving the (re)infusion of living cells, such as hematopoietic stem cell transplantation (HSCT), conceptually differ from ACT. ACT involves the (re-)introduction of a cell population enriched in potentially tumor-reactive immune effectors [23, 24, 81]. HSCT is employed as a means to reconstitute a healthy, allogeneic (and hence potentially tumor-reactive) immune system in patients with hematological malignancies previously subjected to myelo- and lymphoablating treatments (which aim at eradicating the majority of neoplastic cells) [82]. Dendritic cell (DC)-based interventions should also be conceptually differentiated from ACT for two reasons. First, (re-)infused DCs are not endowed with intrinsic anticancer activity, but act as anticancer vaccines to elicit a tumor-targeting immune response [83, 84]. Second, DCs are not administered in the context of lympho/myeloablating chemo(radio)therapy [85-87].

Several strategies have been devised to improve the therapeutic potential of ACT [79, 80, 88]. For instance, genetic engineering has been employed to endow peripheral blood lymphocytes (PBLs) with features such as a unique antigen specificity [89], an increased proliferative potential and persistence *in vivo* [90-93], an improved secretory profile [91], an elevated tumor-infiltrating capacity [94, 95], and superior cytotoxicity [96]. The specificity of PBLs can be altered prior to (re-)infusion by genetically modifying them to express: (1) a TAA-specific T-cell receptor (TCR) [89, 97-99], or (2) a so-called “chimeric antigen receptor” (CAR), i.e., a transmembrane protein comprising the TAA-binding domain of an immunoglobulin linked to one or more immunostimulatory domains [100-106]. The latter approach is advantageous in that it renders T cells capable of recognizing (and hence potentially killing) TAA-expressing cells in an MHC-independent fashion. Several clinical trials have already demonstrated the therapeutic potential of CAR-expressing T cells, in particular (but not only) for patients affected by hematological malignancies [102, 107-111]. T cells expressing TAA-specific TCRs have also been shown to provide objective benefit to cancer patients [89, 97-99]. Conversely, in spite of promising preclinical findings [112-117], the adoptive transfer of purified natural killer (NK) cells to cancer patients has been associated with limited therapeutic activity [118-120]. To the best of our knowledge, the adoptive transfer of purified B lymphocytes has not yet been investigated in the clinic [121], possibly because B cells (or at least some subsets thereof) can exert potent immunosuppressive effects [122-125]. Of note, no ACT protocol is currently approved by the US FDA for use in cancer patients (source http://www.fda.gov).

Since (re-)infused T cells are endowed with intrinsic antineoplastic activity, ACT is generally considered as a passive form of immunotherapy. However, the survival, expansion, migration and cytotoxic activity of adoptively transferred T cells rely on several cytokines, some of which are supplied by the host immune system. Current ACT protocols involve indeed the administration of exogenous interleukins (ILs), including IL-2, IL-15 or IL-21 [126-130], but these stimulate a cytokine cascade in the host that sustains the survival and activity of adoptively transferred cells. Thus, ACT may not represent a *bona fide* paradigm of passive immunotherapy.

#### Oncolytic viruses

The term “oncolytic viruses” refers to non-pathogenic viral strains that specifically infect cancer cells, triggering their demise [131-133]. Oncolytic viruses must be conceptually differentiated from so-called “oncotropic viruses”, i.e., viruses that exhibit a preferential tropism for malignant cells but no (or very limited) cytotoxic activity [134, 135]. The antineoplastic potential of oncolytic viruses can be innate and simply originate from the so-called cytopathic effect, i.e., the lethal overload of cellular metabolism resulting from a productive viral infection [136, 137]. As an alternative, these viruses can mediate an oncolytic activity because of (endogenous or exogenous) gene products that are potentially lethal for the host cell, irrespective of their capacity to massively replicate and cause a cytopathic effect [131, 132]. Of note, genetic engineering has been successfully employed to endow oncolytic virus with various advantageous traits, including sequences coding for (1) enzymes that convert an innocuous pro-drug into a cytotoxic agent [138-143]; (2) proteins that (at least theoretically) trigger lethal signaling cascades in cancer cells only [144-146]; or (3) short-hairpin RNAs that target factors that are strictly required for the survival of transformed, but not normal cells [147, 148]. Of note, no oncolytic virus has been approved by the US FDA for use in cancer patients (source http://www.fda.gov). Conversely, a recombinant adenovirus (H101, commercialized under the name of Oncorine^®^) has been approved by the regulatory authorities of the People's Republic of China for the treatment of HNC (in combination with chemotherapy) as early as in November 2005 [149, 150].

As oncolytic viruses are endowed with intrinsic anticancer activity, they are generally viewed as passive immunotherapeutics. Moreover, several effectors of innate and adaptive immunity limit the efficacy of oncolytic therapy because they can neutralize viral particles before they reach neoplastic lesions [131, 132, 151]. This is particularly true for the mononuclear phagocytic system of the liver and spleen, which is able to sequester large amounts of oncolytic viruses upon injection [152, 153]; the complement system, to which oncolytic viruses are particularly sensitive [154, 155]; and neutralizing antibodies, which can exist in patients prior to oncolytic virotherapy owing to their exposure to naturally occurring variants of the viral strains commonly employed for this purpose [156, 157]. This being said, accumulating preclinical and clinical evidence indicates that the therapeutic activity of oncolytic viruses stems, for the most part, from their ability to elicit tumor-targeting immune responses as they promote the release of TAAs in an immunostimulatory context. In support of this notion, oncolytic viruses engineered to drive the expression of co-stimulatory receptors [158-160] or immunostimulatory cytokines/chemokines [161-165] reportedly mediate superior antineoplastic effects as compared to their unmodified counterparts [131, 132]. Thus, conventional oncolytic viruses also appear to be active, rather than passive, immunotherapeutics.

### Active immunotherapy

#### DC-based immunotherapies

Throughout the past 2 decades, remarkable efforts have been invested in the development of anticancer immunotherapeutics based on (most often autologous) DCs [28, 166, 167]. This intense wave of preclinical and clinical investigation reflects the critical position occupied by DCs at the interface between innate and adaptive immunity, and the ability of some DC subsets to prime robust, therapeutically relevant anticancer immune responses [168]. Several forms of DC-based immunotherapy have been developed, most of which involve the isolation of patient- or donor-derived circulating monocytes and their amplification/differentiation *ex vivo*, invariably in the presence of agents that promote DC maturation, such as granulocyte macrophage colony-stimulating factor (GM-CSF) [28]. This is particularly important because immature DCs exert immunosuppressive, rather than immunostimulatory, functions [169-171]. Most often, autologous DCs are re-infused into cancer patients upon exposure to a source of TAAs, including (1) TAA-derived peptides [172-175]; (2) mRNAs coding for one or more specific TAAs [176]; (3) expression vectors coding for one or more specific TAAs [177-180]; (4) bulk cancer cell lysates (of either autologous or heterologous derivation) [181-186]; (5) or bulk cancer cell-derived mRNA [187-191]. As an alternative, DCs are allowed to fuse *ex vivo* with inactivated cancer cells, generating so-called dendritomes [192-197]. The rationale behind all these approaches is that DCs become loaded *ex vivo* with TAAs or TAA-coding molecules, hence becoming able to prime TAA-targeting immune responses upon reinfusion. Additional DC-based anticancer immunotherapies include the targeting of specific TAAs to DCs *in vivo* [169, 198-205], the use of DC-derived exosomes [206-208], and the (re-)administration of autologous or allogeneic DCs amplified, matured and optionally genetically modified *ex vivo*, but not loaded with TAAs [209-214]. In the former setting, TAAs are fused to mAbs, polypeptides or carbohydrates that selectively bind to DCs [169, 198-202, 215, 216], encapsulated in DC-targeting immunoliposomes [217, 218], or (3) encoded by DC-specific vectors [219-221]. In the latter scenarios, DCs or their exosomes are administered as a relatively non-specific immunostimulatory intervention [209-213]. Interestingly, one cellular product containing a significant proportion of (partially immature) DCs is currently licensed for use in cancer patients, namely sipuleucel-T (also known as Provenge^®^) (source http://www.fda.gov). Sipuleucel-T has been approved by the US FDA and the EMA for the therapy of asymptomatic or minimally symptomatic metastatic castration-refractory prostate cancer as early as in 2010 [222-224]. However, the manufacturer of sipuleucel-T, Dendreon Co. (Seattle, WA, US), filed for bankruptcy in November 2014 (source http://dealbook.nytimes.com/2014/11/10/dendreon-maker-of-prostate-cancer-drug-provenge-files-for-bankruptcy/?_r=0). This reflects the disadvantageous cost-benefit ratio of such a cellular therapy, whose preparation requires a relatively elevated quantity of each patient's peripheral blood mononuclear cells [25, 222, 223]. The safety and efficacy of many DC-based cellular preparations other than are sipuleucel-T are currently being investigated in clinical settings, with promising results [225].

Although DCs isolated from cancer patients have been shown to exert cytotoxic activity against malignant cells [226], DC-based immunotherapies mediate antineoplastic effects mainly because they engage the host immune system against malignant lesions [227, 228]. Thus, all forms of DC-based anticancer interventions constitute paradigms of active immunotherapy.

#### Peptide- and DNA-based anticancer vaccines

DCs and other antigen-presenting cells (APCs) are also targeted by peptide- and DNA-based anticancer vaccines [83, 84, 229-231]. In the former scenario, full-length recombinant TAAs or peptides thereof are administered to cancer patients, most often via the intramuscular, subcutaneous or intradermal route, together with one or more immunostimulatory agents commonly known as adjuvants (which potently promote DC maturation) [232-237]. The rationale behind this approach is that resident DCs (or other APCs) acquire the ability to present the TAA-derived epitopes while maturing, hence priming a robust TAA-specific immune response [32, 238, 239]. The mechanisms underlying the priming of anticancer immune responses by peptide-based vaccines, and hence their efficacy, depend (at least in part) on their size [38]. Thus, while short peptides (8-12 amino acids) are conceived to directly bind to MHC molecules expressed on the surface of APCs, synthetic long peptides (25-30 residues) must be taken up, processed and presented by APCs for eliciting an immune response [38]. Normally, the therapeutic activity of synthetic long peptides is superior to that of their short counterparts, especially when they include epitopes recognized by both cytotoxic and helper T cells or when conjugated to efficient adjuvants [38, 240, 241]. This said, some commonly used immunostimulants such as the so-called incomplete Freund's adjuvant (IFA) have recently been shown to limit the efficacy of peptide-based anticancer vaccination [242], calling for the use of alternative immunostimulants. A peculiar type of peptide-based vaccines is constituted by autologous tumor lysates complexed with immunostimulatory chaperones, most often members of the heat-shock protein (HSP) family [243]. This approach is advantageous in that it does not rely on a single TAA but (at least hypothetically) on all TAAs that bind to HSPs (including patient-specific neo-TAAs) [243]. However, generating anticancer vaccines on a personalized basis is associated with considerable costs [243].

DNA-based anticancer vaccines rely on TAA-coding constructs, be them naked or vectored (by viral particles, non-pathogenic bacteria or yeast cells) [32, 244-246]. DNA-based vaccines either become a source of such TAA (as it is the case for bacterial and yeast vectors) or transform APCs or muscular cells to do so (as it is the case for naked constructs and viral vectors) [32, 244-247]. Theoretically, and especially in the presence of adequate adjuvants, this prompts resident DCs or other APCs to prime a TAA-targeting immune response [32, 183, 248, 249]. A particularly interesting approach in this context is represented by so-called “oncolytic vaccines”, i.e., oncolytic viruses genetically altered to code for a TAA [250-252]. Promising results have also been obtained with DNA-based vaccines administered *per os* [253-256]. In this setting, live-attenuated bacteria expressing a full-length TAA are taken up by APCs in the intestinal mucosa, resulting in the priming of a robust, TAA-specific immune response in the so-called “mucosa-associated lymphoid tissue” [253-256].

Both peptide- and DNA-based vaccines have been associated with clinical activity in patients affected by various neoplasms [83, 84, 229-231, 257]. For instance, a peptide-based vaccine targeting the human papillomavirus type 16 (HPV-16) proteins E6 and E7 have been shown to promote complete, long-lasting responses in a significant fraction of patients with vulvar intraepithelial neoplasia [258]. Along similar lines, the administration of a multipeptide vaccine after single-dose cyclophosphamide (an immunogenic alkylating agent, see below) has been shown to prolong overall survival in a cohort of RCC patients [259]. No peptide- or DNA-based anticancer vaccine is currently approved by the US FDA and EMA for use in humans (sources http://www.fda.gov and http://www.ema.europa.eu/ema/). However, vitespen (Oncophage^®^), a heat shock protein 90kDa beta (Grp94), member 1 (HSP90B1)-based anticancer vaccine, has been approved in Russia for the treatment of RCC patients with intermediate risk of recurrence as early as in 2008 [257]. Moreover, three DNA-based anticancer vaccines have been licensed for veterinary use [260-263], one of which relies on a human TAA (*i.e.*, tyrosinase) [263].

Similar to DC-based interventions, both peptide- and DNA-based anticancer vaccines mediate antineoplastic effects as they (re-)activate the host immune system against malignant cells, hence constituting active forms of anticancer immunotherapy.

#### Immunostimulatory cytokines

Taken as a family, cytokines regulate (via autocrine, paracrine or endocrine circuits) virtually all biological functions [264-267]. It is therefore not surprising that various attempts have been made to harness the biological potency of specific cytokines to elicit novel or reinvigorate pre-existent tumor-targeting immune responses [268-271]. The administration of most immunostimulatory cytokines to cancer patients as standalone therapeutic interventions, however, is generally associated with little, if any, clinical activity [272-275]. Thus, immunostimulatory cytokines are generally employed as adjuvants for other anticancer (immuno)therapeutics, either as recombinant molecules or encoded within expression vectors [276-284]. Notable exceptions include interferon (IFN)-α2b (also known as Intron A^®^), and IL-2 (also known as aldesleukin and Proleukin^®^), which mediate single agent therapeutic activity in patients affected by melanoma, a tumor type particularly sensitive to immunotherapy [274, 284]. IFN-α2b is currently approved by the US FDA and EMA for the therapy of hairy cell leukemia (HCL), AIDS-related Kaposi's sarcoma, follicular lymphoma, multiple myeloma, melanoma, external genital/perianal warts (*condylomata acuminata*) and cervical intraepithelial neoplasms (both as a recombinant, unmodified protein, and as a pegylated variant), while IL-2 is licensed for the treatment of metastatic forms of melanoma and RCC. Moreover, IFN-α2a (also known as Roferon-A^®^) is approved for use in subjects with HCL and chronic phase, Philadelphia chromosome-positive chronic myeloid leukemia, upon minimal pretreatment (within 1 year of diagnosis). In Europe, IFN-α2a is also licensed for the treatment of melanoma. Of note, GM-CSF (also known as molgramostim, sargramostim, Leukomax^®^, Mielogen^®^ or Leukine^®^) and granulocyte colony-stimulating factor (G-CSF, also known as filgrastim, lenograstim or Neupogen^®^) are approved by the US FDA and EMA for use in humans, but not as part of anticancer regimens [285-288]. Nonetheless, GM-CSF has been shown to potentiate the clinical activity of several immunotherapeutics, including (but not limited to) peptide-based vaccines and immunomodulatory mAbs [259, 289]. Recombinant tumor necrosis factor α (TNFα) is also licensed by several regulatory agencies worldwide (but not by the US FDA), for the treatment of limb-threatening soft tissue sarcoma and melanoma [290-292]. However, in this setting TNFα is not employed as an immunostimulatory agent but administered in combination with melphalan (an alkylating agent) to increment the local concentration of the drug (and hence boost its cytotoxicity), and to promote the selective destruction of the tumor vasculature [293].

The antineoplastic activity of immunostimulatory cytokines is expected to depend on the host immune system, implying that they underlie a *bona fide* paradigm of active immunotherapy. However, the actual mode of action of immunostimulatory cytokines has not yet been fully explored. Moreover, some of these agents may promote a cytokine cascade with unwarranted, potentially lethal effects, and hence should be employed with caution.

#### Immunomodulatory mAbs

At odds with their tumor-targeting counterparts, immunomodulatory mAbs operate by interacting with (hence altering the function of) soluble or cellular components of the immune system [22, 294]. Thus, immunomodulatory mAbs are designed to elicit a novel or reinstate an existing anticancer immune response [27, 295, 296]. So far, this has been achieved through four general strategies: (1) the inhibition of immunosuppressive receptors expressed by activated T lymphocytes, such as cytotoxic T lymphocyte-associated protein 4 (CTLA4) [297-299] and programmed cell death 1 (PDCD1, best known as PD-1) [39, 42, 300, 301], or NK cells, like various members of the killer cell immunoglobulin-like receptor (KIR) family [302-304]; (2) the inhibition of the principal ligands of these receptors, such as the PD-1 ligand CD274 (best known as PD-L1 or B7-H1) [300, 305-307]; (3) the activation of co-stimulatory receptors expressed on the surface of immune effector cells [308] such as tumor necrosis factor receptor superfamily, member 4 (TNFRSF4, best known as OX40) [309-313], TNFRSF9 (best known as CD137 or 4-1BB) [58, 314, 315], and TNFRSF18 (best known as GITR) [316-318]; and (4) the neutralization of immunosuppressive factors released in the tumor microenvironment, such as transforming growth factor β1 (TGFβ1) [319, 320].

The first of these approaches, which is commonly referred to as “checkpoint blockade”, has been shown to induce robust and durable responses in cohorts of patients with a variety of solid tumors [39, 300, 321-327]. As it stands, no less than three checkpoint-blocking mAbs are currently approved by international regulatory agencies for use in humans (source http://www.fda.gov): (1) the anti-CTLA4 mAb ipilimumab (Yervoy™), which was licensed by the US FDA for use in individuals with unresectable or metastatic melanoma on 2011, March 25^th^ [328-332]; the anti-PD-1 mAb pembrolizumab (Keytruda™), which received accelerated approval by the US FDA for the treatment of advanced or unresectable melanoma patients who fail to respond to other therapies on 2014, September 4^th^ [333-338]; and nivolumab (Opvido™), another PD-1-targeting mAb licensed by the Japanese Ministry of Health and Welfare for use in humans on 2014, July 07^th^ [339]. Based on the results of a recently completed Phase III clinical trial demonstrating that nivolumab significantly improves the progression-free and overall survival of patients with BRAF^WT^ melanoma [340], the approval of this mAb by the US FDA is expected within the next few months. The safety and efficacy of ipilimumab, pembrolizumab, nivolumab and other checkpoint-blocking mAbs are being demonstrated in a steadily expanding panel of oncological indications [45, 46, 341, 342]. Of note, some co-stimulatory mAbs including urelumab and PF-0582566 (both of which target CD137) are also under clinical development, with promising results [46, 341]. Preclinical data suggest that combining checkpoint blockers with co-stimulatory mAb mediates superior antineoplastic effects [294, 343, 344]. At least in part, this reflects the ability of co-stimulatory mAbs to promote NK cell functions [58, 345, 346]. In line with this notion, a few clinical trials testing checkpoint blockers in combination with urelumab or lirilumab (a KIR-inhibiting mAb) have just been initiated (source http://www.clinicaltrials.gov).

Designed to (re-)activate the host immune system against malignant cells, immunomodulatory mAbs constitute an established and clinically promising paradigm of active immunotherapy. Interestingly, despite their non-specific mechanism of action, the clinical efficacy of immunomodulatory mAbs (and in particular checkpoint blockers) may be profoundly influenced by the panel of (neo-)TAAs specific to each neoplasm [347].

#### Inhibitors of immunosuppressive metabolism

Indoleamine 2,3-dioxigenase 1 (IDO1) catalyzes the first, rate-limiting step in the so-called “kynurenine pathway”, the catabolic cascade that converts *L*-tryptophan (Trp) into *L*-kynurenine (Kyn) [348]. Although this enzyme was initially believed to mediate immunostimulatory effects (partly because inflammatory cues including IFNγ promote its expression in cells of the innate immune system) [349, 350], IDO1 mediates robust immunosuppressive effects, in both physiological (*e.g.*, tolerance during pregnancy) and pathological (mostly oncological) settings [351-356]. IDO1 has been proposed to inhibit both innate and adaptive immune responses (1) by depleting immune effector cells of Trp, resulting in irresponsiveness to immunological challenges [352, 353, 357-359]; (2) by favoring the accumulation of Kyn and some of its derivatives, which exert cytotoxic effects on immune effector cells while promoting the differentiation of Tregs [360-364]; or (3) through various indirect mechanisms mediated by IDO1-expressing DCs [124, 365-371]. Evidence accumulated during the last decade indicates that both 1-methyltryptophan (an inhibitor of IDO1 and IDO2) and genetic interventions targeting IDO1 mediate antineoplastic effects while eliciting novel or reinvigorating existent anticancer immune responses [372-375]. No IDO1 inhibitor is currently approved by the US FDA for use in humans (source http://www.fda.gov). However, the results of recent Phase I-II studies suggest that 1-methyl-*D*-tryptophan (an inhibitor of the IDO pathway also known as indoximod), other pharmacological blockers of IDO1 (such as INCB024360), and IDO1-targeting vaccines are well tolerated by cancer patients and mediate antineoplastic effects, at least in a subset of individuals [376-382].

Extracellular ATP mediates robust immunostimulatory functions as it recruits and activates APCs via purinergic receptor P2Y, G-protein coupled, 2 (P2RY2) and purinergic receptor P2X, ligand-gated ion channel, 7 (P2RX7), respectively [383-386]. On the contrary, the degradation products of ATP (notably AMP and adenosine), have a pronounced immunosuppressive activity upon binding to adenosine A2a receptor (ADORA2A) and ADORA2B [387-389]. Two enzymes operates sequentially to degrade extracellular ATP, ectonucleoside triphosphate diphosphohydrolase 1 (ENTPD1, best known as CD39), which converts ATP into ADP and AMP [390-392], and 5′-nucleotidase, ecto (NT5E, best known as CD73), which transforms AMP into adenosine [393, 394]. Some human neoplasms express increased amounts of CD39 and/or CD73, reflecting the evolutionary advantage conferred to cancer cells by the stimulation of adenosine receptors [395, 396]. Efforts have therefore been dedicated to the development of agents that would limit the extracellular availability of adenosine or inhibit adenosine receptors [392, 397]. Preclinical evidence indicates that CD39- or CD79-targeting agents (mostly mAbs) mediate antineoplastic effects as standalone interventions and improve the efficacy of other anticancer agents [397]. The clinical development of these agents, however, has not yet been initiated. Conversely, ADORA2A antagonists are currently being tested in late-stage clinical trials, but as a therapeutic option against Parkinsonism [397]. It will be interesting to determine the safety and efficacy of inhibitors of adenosine generation or signaling in cancer patients.

Although it remains unclear whether these agents truly operate by altering the microenvironmental availability of Trp and Kyn [398], the antineoplastic effects of IDO inhibitors critically rely on the host immune system, implying that this constitutes an instance of active anticancer immunotherapy [399]. This also applies to strategies aimed at limiting the extracellular availability of adenosine.

#### PRR agonists

Pattern recognition receptors (PRRs) are evolutionarily conserved proteins involved in the recognition of danger signals [400, 401]. PRRs include (but are not limited to) Toll-like receptors (TLRs) [402, 403] and nucleotide-binding oligomerization domain containing (NOD)-like receptors (NLRs) [404, 405]. TLRs are transmembrane enzymatically-inactive proteins expressed by most APCs, including monocytes, macrophages and DCs, as well as by some types of epithelial cells [402, 403]. NLRs are expressed by a variety of cell types, including various components of the innate and adaptive immune system [404, 405]. Taken together, PRRs sense a wide panel of danger signals, including exogenous “microbe-associated molecular patterns” (MAMPs) like bacterial lipopolysaccharide (LPS) or muramyl dipeptide (MDP), and endogenous “damage-associated molecular patterns” (DAMPs), like the non-histone nuclear protein high-mobility group box 1 (HMGB1) and mitochondrial DNA [406-410]. The activation of various PRRs ignites a signal transduction cascade with potent pro-inflammatory outcomes, including the activation of NF-κB [411-413], and the secretion of immunostimulatory cytokines, like type I IFNs and TNFα [413-415]. Moreover, PRR signaling favors the maturation of DCs as well as the activation of macrophages and NK cells [416]. Besides being critical for the response of the host to viral and bacterial challenges [402, 403], some PRRs play a key role in the (re)activation of anticancer immune responses by chemo-, radio- and immunotherapeutic interventions [15, 413, 417-422].

Thus, PRR agonists have spurred interest not only as adjuvants for conventional vaccines [423, 424], but also as immunotherapeutic interventions that may mediate antineoplastic effects *per se* or boost the therapeutic activity of other anticancer agents [34, 48, 425]. Three TLR agonists are approved by the US FDA for use in cancer patients: (1) the bacillus Calmette-Guérin (BCG), an attenuated variant of *Mycobacterium bovis* that presumably operates as a mixed TLR2/TLR4 agonist, which is currently used as a standalone immunotherapeutic agent in subjects with non-invasive transitional cell carcinoma of the bladder [426]; (2) monophosphoryl lipid A (MPL), a TLR2/TLR4-activating derivative of *Salmonella minnesota* LPS currently utilized as adjuvant in Cervarix^®^, a vaccine for the prevention of HPV-16 and -18 infection [427]; and (3) imiquimod, an imidazoquinoline derivative that triggers TLR7 signaling, currently employed for the treatment of actinic keratosis, superficial basal cell carcinoma and *condylomata acuminata* [422, 426]. Of note, picibanil (a lyophilized preparation of *Streptococcus pyogenes* that operates as a TLR2/TLR4 agonist has been licensed for use in cancer patients by the Japanese Ministry of Health and Welfare (but not by the US FDA) as early as in 1975 [428, 429]; while mifamurtide (a synthetic lipophilic glycopeptide that activates NOD2) has been approved by the EMA for the treatment of osteosarcoma in 2009 [430-432]. Moreover, the safety and efficacy of several other PRR agonists are currently being evaluated in clinical trials [433-435]. These molecules include agatolimod (CpG-7909, PF-3512676, Promune^®^), an unmethylated CpG oligodeoxynucleotide that activates TLR9 [436]; polyriboinosinic polyribocytidylic acid (polyI:C, Ampligen™, Rintatolimod), a synthetic double-strand RNA that signals via TLR3 [437]; and Hiltonol™, a particular formulation of polyI:C that involves carboxymethylcellulose and poly-*L*-lysine [48, 438].

Some malignant cells express PRRs [439-445], implying that PRR agonists may not be completely devoid of intrinsic tumor-modulating functions. Nonetheless, a large body of preclinical and clinical literature indicates that the antineoplastic effects of PRR agonists stem from their ability to engage the host immune system. Thus, PRR agonists constitute active immunotherapeutics.

#### Immunogenic cell death inducers

Some conventional chemotherapeutics, often employed at metronomic doses [446, 447], as well as some forms of radiation therapy, can kill malignant cells while stimulating them to release specific DAMPs in a spatiotemporally coordinated manner [15, 420, 448]. Such DAMPs bind to receptors expressed on the surface of APCs (including TLR4), and not only boost their ability to engulf particulate material (including TAAs and cancer cell debris) but also trigger their maturation/activation [15, 418, 448, 449]. As a result, APCs acquire the ability to elicit a cancer-specific immune response that (at least in mice) is associated with the development of immunological memory [15, 450]. We have dubbed such a functionally atypical form of apoptosis “immunogenic cell death” (ICD) [15]. Importantly, ICD inducers exert optimal antineoplastic effects in immunocompetent, but not in immunodeficient, mice [15, 451-454]. However, the ability of a specific stimulus to trigger ICD can be properly assessed only by means of vaccination experiments involving immunocompetent mice and syngeneic tumor models [15, 455]. As it stands, a few FDA-approved therapies have been shown to constitute *bona fide* ICD inducers, including: doxorubicin, mitoxantrone and epirubicin (three anthracyclines currently employed against various carcinomas) [186, 449], bleomycin (a glycopeptide antibiotic endowed with antineoplastic properties) [456], oxaliplatin (a platinum derivative generally used for the therapy of colorectal carcinoma) [453, 457], cyclophosphamide (an alkylating agent employed against neoplastic and autoimmune conditions) [458-460], specific forms of radiation therapy [419, 461-466], photodynamic therapy (an intervention that relies on the administration of a photosensitizing agent coupled to light irradiation) [448, 467, 468], and bortezomib (a proteasomal inhibitor used for the treatment of multiple myeloma) [469, 470].

These and other (hitherto experimental) ICD inducers have been viewed as conventional forms of anticancer therapy, exerting antineoplastic effects via cytostatic or cytotoxic mechanisms. However, accumulating evidence indicates that the full-blown therapeutic potential of these molecules relies on the host immune system [15, 471]. Thus, we propose to classify ICD inducers as a form of active anticancer immunotherapy.

#### Others

Other anticancer immunotherapies are approved by regulatory agencies worldwide for use in cancer patients or are currently being investigated for safety and efficacy in preclinical or clinical settings.

Lenalidomide (Revlimid^®^, also known as CC-5013) and pomalidomide (Pomalyst^®^, also known as CC-4047) are two derivatives of thalidomide (Thalomid^®^) originally developed in the 1990s to achieve improved potency in the absence of significant side effects [472]. Thalidomide was indeed marketed as an over-the-counter sedative, tranquilizer, and antiemetic for morning sickness in various countries in the late 1950s, but was rapidly withdrawn following a peak of infants born with malformation of the limbs [473]. In spite of its pronounced teratogenic activity, thalidomide raised renewed interest as an inhibitor of TNFα secretion in the 1990s [474], and was approved by the US FDA (under a strictly controlled distribution program) for the therapy of erythema nodosum leprosum (a complication of leprosy etiologically linked to TNFα) in 1998 [475]. The combination of thalidomide with dexamethasone (a glucocorticoid) rapidly turned out to mediate therapeutic effects in patients with hematological malignancies, eventually resulting in the approval by the US FDA of this regimen for the treatment of newly diagnosed multiple myeloma [476]. Alongside, lenalidomide (which retains some degree of teratogenicity) was licensed for use in patients with multiple myeloma (also in combination with dexamethasone) and low or intermediate-1 risk myelodysplastic syndromes that harbor 5q cytogenetic abnormalities (as a standalone intervention) [477-480]. Conversely, pomalidomide (which is devoid of teratogenic activity) has been approved for use in multiple myeloma patients only in 2013, when the approval of lenalidomide has been extended to mantle cell lymphoma (MCL) [481-483]. Although the effects of thalidomide, lenalidomide and pomalidomide, which are collectively referred to as “immunomodulatory drugs” (IMiDs), on the immune system have been characterized with increasing precision throughout the past two decades [484], the underlying molecular mechanisms remained obscure [485]. Recent findings indicate that the therapeutic activity of IMiDs depend, at least in part, on their ability to bind the E3 ubiquitin ligase cereblon (CRBN) and hence boost the proteasomal degradation of the B cell-specific transcription factors IKAROS family zinc finger 1 (IKZF1) and IKZF3 [486, 487]. Of note, CRBN, which is also involved in the teratogenic effects of thalidomide and lenalidomide [488], regulates the abundance of interferon regulatory factor 4, perhaps accounting for the immunomodulatory functions of IMiDs [489]. Although endowed with intrinsic antineoplastic activity, IMiDs should be considered active immunotherapeutics.

As they progress and respond to treatment, neoplastic lesions are infiltrated by a significant amount of lymphoid and myeloid cells, including CD8^+^ T lymphocytes, Tregs, tumor-associated macrophages (TAMs) and immunosuppressive B-cell populations [122-124, 490, 491]. Robust tumor infiltration by CD8^+^ T lymphocytes is generally associated with a good prognosis, especially when the intratumoral levels of Tregs are limited [124, 492]. Along similar lines, high intratumoral levels of TAMs with a “classically-activated” M1 phenotype (which exert tumoricidal functions, stimulate NK cells and secrete T_H_1-polarizing cytokines) generally correlate with improved disease outcome [491, 493]. The contrary holds true when the myeloid tumor infiltrate contains high levels of “alternatively-activated” M2 TAMs or specific B-cell subsets, which can secrete not only immunosuppressive cytokines like IL-10 and TGFβ1, but also angiogenic mediators such as VEGFA and enzymes that remodel the extracellular matrix [491, 493]. These observations prompted the development of immunotherapeutic regimens based on the depletion/inhibition of Tregs or B lymphocytes, as well as on the conversion of M2 TAMs to their M1 counterparts.

Denileukin diftitox (also known as Ontak^®^) is a recombinant variant of IL-2 fused to the diphtheria toxin [494]. Owing to its selective cytotoxicity for cells expressing IL-2 receptor α (IL2RA, best known as CD25), denileukin diftitox has been approved by the US FDA and EMA for the treatment of CD25^+^ cutaneous T-cell lymphoma in the early 2000s [494]. More recently, denileukin diftitox has been tested for its ability to improve the efficacy of various immunotherapies by efficiently depleting Tregs (which also express CD25) in patients affected by various neoplasms [495-497]. In some (but not all) these clinical settings, denileukin diftitox enhanced the efficacy of immunotherapy as it provoked a sizeable Treg depletion [496, 497]. However, denileukin diftitox has recently been ascribed with a number of immunosuppressive effects [498, 499]. This may explain why in some cases denileukin diftitox had no clinical activity [495], and casts doubts on the possibility to use such Treg-depleting agent as a routine anticancer immunotherapeutic. This said, several conventional antineoplastic agents commonly used in the clinic appear to deplete or inhibit Treg, which presumably contributes to their therapeutic activity (see below) [420, 421]. Along similar lines, at least part of the clinical activity of ibrutinib (PCI-32765), a small molecule inhibitor of bruton tyrosine kinase (BTK) recently approved by the US FDA for use in patients with MCL and CLL [500-502], may stem from its ability to target tumor-infiltrating B lymphocytes or myeloid cells [503]. A clinical trial testing this possibility in pancreatic cancer patients will soon be initiated (LC, personal communication).

Several immunotherapeutic agents exert antineoplastic effects by altering the relative proportion between M2 and M1 TAMs in favor of the latter [491]. These include: (1) tasquinimod, a second-generation orally active quinoline-3-carboxamide analog initially developed as an antiangiogenic agent [504, 505]; trabectedin (Yondelis^®^), a marine antineoplastic agent currently approved in Europe, Russia and South Korea for the treatment of soft tissue sarcoma and ovarian carcinoma [506, 507]; (3) inhibitors of chemokine (C-C motif) ligand 2/chemokine (C-C motif) receptor 2 (CCL2/CCR2) signaling [508]; (3) mAbs specific for chemokine (C-X-C motif) receptor 4 (CXCR4) [509]; and (4) small molecule inhibitors and mAbs that suppress colony stimulating factor 1/colony stimulating factor 1 receptor (CSF1/CSFR1) signaling [510-512]. With the single exception of trabectedin (which was not developed as an immunotherapeutic agent), none of these strategies is currently approved by the US FDA or EMA for use in humans (sources http://www.fda.gov and http://www.ema.europa.eu/ema/). However, several Phase II-III clinical trials are currently ongoing to establish the safety and efficacy of these active immunotherapeutic agents in patients with various solid tumors (source http://www.clinicaltrials.gov).

Additional, hitherto experimental immunotherapeutic regimens act by stimulating the host immune system to mount a novel (or unleash an existing) immune response against malignant cells. These include: (1) strategies for the depletion of circulating myeloid-derived suppressor cells (MDSCs), a blood-borne population of immature, immunosuppressive myeloid cells that generally accumulate in the course of tumor progression [513-516]; (2) mAbs that block CD47, one of the major antiphagocytic receptor expressed by malignant cells [517-519]; and (3) vaccines relying on the administration of cancer cell lines expressing immunostimulatory molecules (e.g., GM-CSF) upon inactivation or lysis [520].

## CONCLUDING REMARKS

During the past three decades, immunotherapy has become a clinical reality [35, 78, 521], and an ever-increasing number of cancer patients are expected to receive, at some stage of their disease, an immunotherapeutic intervention [522, 523]. The observations presented above suggest that various immunotherapies previously classified as passive, including several (if not all) tumor-targeting mAbs, ACT and oncolytic viruses, may *de facto* constitute active forms of immunotherapy. Moreover, accumulating preclinical and clinical evidence indicates that therapeutically relevant anticancer immune responses invariably exhibit some degree of epitope spreading, i.e., they eventually target several TAAs even when they were initially directed against a single one [524, 525]. This is not surprising considering that malignant cells exhibit a high degree of genetic/genomic instability and hence are relatively prone to generate so-called “antigen loss variants” that would render TAA-specific immunotherapies completely ineffective with time [526-528]. Thus, even if immunotherapies that truly generate an anticancer response with a unique antigen specificity existed [529, 530], they presumably would not mediate clinically relevant, long-term immune responses. In turn, this casts some doubts on the practical utility of classifying immunotherapies into “antigen-specific” or “non-specific”.

Recently, great attention has been given to the immunostimulatory effects of conventional chemotherapeutics [420, 421, 531, 532]. Indeed, several compounds that have been successfully used in the clinic, including the nucleoside analogs gemcitabine (which is approved by the US FDA for the treatment of pancreatic, ovarian, breast and non-small cell carcinoma) [533, 534] and 5-fluorouracil (which is licensed for use in patients affected by various neoplasms) [535, 536] have off-target immunostimulatory effects, in particular when administered as low doses and according to metronomic schedules (while, similar to radiation therapy, they are generally immunosuppressive when given at high doses) [537, 538]. It is therefore tempting to speculate that most (if not all) anticancer agents that are truly beneficial to patients operate as active immunotherapeutics, stimulating the host immune system to mount an antigenically broad (and hence insensitive to antigen loss) response against malignant cells. In support of this notion, an ever increasing number of combinatorial immuno(chemo)therapeutic regimens is being designed and tested in clinical trials, with promising results [34]. This being said, only the adequate implementation of protocols to monitor immune system-related parameters among patients participating in clinical trials (immunomonitoring) will provide insights into this possibility [539-543]. Such protocols are inherently complex, calling for international efforts toward standardization [544]. Harmonized immunomonitoring procedures will undoubtedly guide the development of new (immuno)therapies, and facilitate the identification of novel prognostic or predictive biomarkers [544]. We are positive that the next clinical success of anticancer immunotherapy is just behind the door.

## Abbreviations

ACT: adoptive cell transfer
ADCC: antibody-dependent cell-mediated cytotoxicity
ADORA: adenosine receptor
APC: antigen-presenting cell
BiTE: bispecific T-cell engager
CAR: chimeric antigen receptor
CLL: chronic lymphocytic leukemia
CRBN: cereblon
CRC: colorectal carcinoma
CTLA4: cytotoxic T lymphocyte-associated protein 4
DAMP: damage-associated molecular pattern
DC: dendritic cell
EGFR: epidermal growth factor receptor
EMA: European Medicines Agency
FCGR2A: Fc fragment of IgG, low affinity IIa, receptor
FCGR3A: Fc fragment of IgG, low affinity IIIa, receptor
FDA: Food and Drug Administration
GM-CSF: granulocyte macrophage colony-stimulating factor
HCL: hairy cell leukemia
HNC: head and neck cancer
HPV: human papillomavirus
HSCT: hematopoietic stem cell transplantation
HSP: heat shock protein
ICD: immunogenic cell death
IDO1: indoleamine 2,3-dioxigenase 1
IFN: interferon
IKZF: IKAROS family zinc finger
IL: interleukin
IMiD: immunomodulatory drug
KIR: killer cell immunoglobulin-like receptor
Kyn: *L*-kynurenine
LPS: lipopolysaccharide
mAb: monoclonal antibody
MCL: mantle cell lymphoma
NLR: NOD-like receptors
NK: natural killer
PBL: peripheral blood lymphocyte
PRR: pattern recognition receptor
RCC: renal cell carcinoma
TAA: tumor-associated antigen
TAM: tumor-associated macrophage
TCR: T-cell receptor
TGFß1: transforming growth factor ß1
TLR: Toll-like receptor
TNFa: tumor necrosis factor a
TNFRSF: tumor necrosis factor receptor superfamily
Treg: regulatory T cell
Trp: tryptophan
VEGFA: vascular endothelial growth factor A
